# Fecal Microbiota Transplantation in Allogeneic Hematopoietic Stem Cell Transplantation Recipients: A Systematic Review

**DOI:** 10.3390/jpm11020100

**Published:** 2021-02-04

**Authors:** Andrea Pession, Daniele Zama, Edoardo Muratore, Davide Leardini, Davide Gori, Federica Guaraldi, Arcangelo Prete, Silvia Turroni, Patrizia Brigidi, Riccardo Masetti

**Affiliations:** 1 Pediatric Oncology and Hematology “Lalla Seràgnoli”, Pediatric Unit—IRCCS Azienda Ospedaliero-Universitaria di Bologna, 40138 Bologna, Italy; andrea.pession@unibo.it (A.P.); daniele.zama@aosp.bo.it (D.Z.); davide.leardini3@gmail.com (D.L.); arcangelo.prete@aosp.bo.it (A.P.); riccardo.masetti5@unibo.it (R.M.); 2 Department of Biomedical and Neuromotor Sciences (DIBINEM), University of Bologna, 40126 Bologna, Italy; davide.gori4@unibo.it (D.G.); federica.guaraldi@yahoo.it (F.G.); 3 IRCCS Istituto delle Scienze Neurologiche di Bologna, 40126 Bologna, Italy; 4 Department of Pharmacy and Biotechnology (FABIT), University of Bologna, 40126 Bologna, Italy; silvia.turroni@unibo.it; 5 Department of Medical and Surgical Sciences (DIMEC), University of Bologna, 40126 Bologna, Italy; patrizia.brigidi@unibo.it

**Keywords:** hematopoietic stem cell transplantation, fecal microbiota transplantation, gut microbiota, aGvHD, antibiotic-resistant bacteria

## Abstract

The disruption of gut microbiota eubiosis has been linked to major complications in allogeneic hematopoietic stem cell transplantation (allo-HSCT) recipients. Various strategies have been developed to reduce dysbiosis and related complications. Fecal microbiota transplantation (FMT) consists of the infusion of fecal matter from a healthy donor to restore impaired intestinal homeostasis, and could be applied in the allo-HSCT setting. We conducted a systematic review of studies addressing the use of FMT in allo-HSCT patients. In the 23 papers included in the qualitative synthesis, FMT was used for the treatment of recurrent *Clostridioides difficile* infections or as a therapeutic strategy for steroid-resistant gut aGvHD. FMT was also performed with a preventive aim (e.g., to decolonize from antibiotic-resistant bacteria). Additional knowledge on the biological mechanisms underlying clinical findings is needed in order to employ FMT in clinical practice. There is also concern regarding the administration of microbial consortia in immune-compromised patients with altered gut permeability. Therefore, the safety profile and efficacy of the procedure must be determined to better assess the role of FMT in allo-HSCT recipients.

## 1. Introduction

Allogeneic hematopoietic stem cell transplantation (allo-HSCT) is a potential curative strategy for many oncological, hematological, metabolic and immunological diseases [1,2,3]. Despite advances in transplantation technology and supportive care, the procedure is still associated with marked morbidity and mortality, mainly due to the recurrence of the primary disease or transplant-related complications [4]. Infections and acute Graft versus Host Disease (aGvHD) represent two of the main transplant-related complications after allo-HSCT [5].

Chemo and radiotherapy prior to transplant ablate circulating white blood cells and damage the gut epithelium, enabling the translocation of microbes through the intestinal mucosa and eventually into the bloodstream [6]. Therefore, potentially life-threatening bacterial infections can occur during the early neutropenic post-transplant phase, with the burden of antibiotic-resistant bacteria (ARB) being a critical issue in the management of these patients [7].

aGvHD is characterized by the response of alloreactive donor T cells to host organs including the skin, gut and liver. Multiple signals interact with lymphocytes and antigen-presenting cells to regulate the allo-immune response, such as the level of inflammatory cytokines and the presence of damage- and pathogen-associated molecular patterns [8]. Corticosteroids represent the first line therapy for aGvHD treatment, but their administration results in complete remission in less than half of the patients [9,10]. Over the last few years, numerous novel agents have been developed and investigated for the management of steroid-refractory or steroid-dependent disease, but no definitive consensus has been reached on the optimal second-line therapy for aGvHD [9,10].

Among the numerous factors known to be involved in the development of these complications, the recipient gut microbiome (GM) is emerging as a key determinant. The advent of large-scale genomic sequencing studies has greatly improved our ability to characterize the complex microbial communities hosted by our organism and enhanced our comprehension of the relationship between GM, immunity and intestinal epithelium [11,12]. In particular, the GM is recognized as an integral part of the host immune system, capable of fine-tuning immune responses, thus strongly contributing to homeostasis. Moreover, through the production of a plethora of bioactive molecules, the GM may also signal to various extraintestinal organs, having a system-level impact on human health. In this regard, the increasing use of the so-called omics approaches, including metagenomics, metatranscriptomics, metaproteomics, metabolomics and not least, culturomics, is starting to shed some light on the biological processes underpinning the crosstalk between the GM and the host [7,11].

HSCT and related procedures (i.e., conditioning regimen, antibiotic exposure, diet, antiacid prophylaxis) represent a combination of upsetting events that profoundly modifies the GM structure, leading to disruption of the mutualistic asset, with the establishment of the so-called dysbiosis [13,14]. Lower alpha diversity of the GM at the time of neutrophil engraftment was associated with higher transplantation-related mortality and lower overall survival [15]. Moreover, specific GM compositional layouts were associated with clinical allo-HSCT outcomes. For example, decreased amounts of beneficial bacteria belonging to the order Clostridiales (e.g., the genus *Blautia*) and a shift towards an enteropathogenic community with predominance of Gram-negative Enterobacteriales (*Escherichia coli*, *Klebsiella*, *Enterobacter* spp.) along with Gram-positive Lactobacillales (*Lactobacillus*, *Enterococcus* and *Streptococcus* spp.) were correlated with increased incidence of aGvHD and aGvHD-related mortality [16,17]. Intestinal dominance by individual taxa, defined as a single bacterial taxon comprising 30% or more of the GM, often precedes the development of a corresponding bloodstream infection [18]. However, the main limitation of these studies is their observational nature, and so they can only demonstrate correlations and not causative relationships.

This increasing knowledge on the GM role in the pathophysiology of the main allo-HSCT complications has led to fascinating ideas for modulating the intestinal ecosystem in order to improve clinical outcomes. Recently, numerous therapeutic strategies have been proposed in the literature to prevent the damage or restore GM integrity, including the optimization of antibiotic administration [19], the route of nutritional support [20,21,22] and the use of prebiotics [23]. GM can also be modulated using live microorganisms or microbial consortia, from traditional probiotics or next-generation candidates to fecal microbiota transplantation (FMT). FMT consists of the infusion of fecal matter from a healthy donor into the gastrointestinal tract of a recipient harboring a dysbiotic GM. The source of fecal material could be autologous, with stools collected before the onset of dysbiosis, or from a related or unrelated healthy donor. Because of genetic similarity and shared environment, a related FMT donor may have a closer GM composition, which may be inadvisable in certain cases. Stools can be handled and prepared as fresh fecal material, or frozen and stored in a stool bank. FMT can be delivered via the upper gastrointestinal tract using esophagogastroduodenoscopy, nasogastric or nasoduodenal tube and oral capsule, or via colonoscopy and enema [24]. This procedure directly modifies the host GM composition in an attempt to restore GM diversity and gut homeostasis [25]. FMT was first shown to be successful in the treatment of recurrent *Clostridioides difficile* infections (rCDI) and is now recommended in patients with rCDI in whom appropriate antibiotic treatments failed [26]. Thanks to its potential to re-establish an eubiotic GM layout in the recipient, FMT has been proposed for the treatment of other conditions, including inflammatory bowel disease, with promising preliminary findings [27].

In this context, there is a growing interest for FMT in allo-HSCT as a potential preventive or therapeutic strategy, mainly regarding aGvHD and infections [28]. However, many practical and safety issues arise in this setting, which have limited its application in recent years. Different FMT protocols could be applied, varying with regards to donor selection and screening, preparation of recipients and route of infusion [24]. Safety concerns have been raised regarding its use in immune-compromised patients with impaired gut permeability [29]. Indeed, a case of bacteremia caused by a multidrug-resistant *E*. *coli* transmitted through FMT has recently been reported, which led to the patient’s death [30].

Numerous publications have reviewed this topic, either as the main focus of the paper or as a part of a more comprehensive view on the role of GM in transplantation, but this is the first systematic review on this issue. The aim of this study is to provide an up-to-date systematic review regarding the evolving evidence on the use of FMT in allo-HSCT recipients, summarizing the present literature and providing insights for future investigations.

## 2. Methods

A systematic review was conducted according to the Preferred Reporting Items for Systematic Reviews and Meta-Analyses (PRISMA) guidelines [31]. Electronic databases, including PubMed (https://pubmed.ncbi.nlm.nih.gov) and Trip (https://www.tripdatabase.com) were searched to identify relevant studies published up to October 2020. The following string was used to perform the literature search: (Bone Marrow transplant * OR BMT OR stem cell transplant * OR SCT OR hematopoietic transplant * OR haematopoietic transplant * OR hematopoietic stem cell transplant * OR haematopoietic stem cell transplant * OR hematopoietic cell transplant * OR haematopoietic cell transplant * OR HCT OR HSCT OR blood disorders OR leukemia OR immunocompromised) AND (fecal microbiota transplant * OR faecal microbiota transplant * OR FMT).

The search was restricted to English-language studies involving human subjects undergoing allo-HSCT receiving FMT for any indication. Two reviewers (EM, DL) independently identified potentially eligible studies by screening titles and abstracts. The same authors assessed the full-texts of potentially relevant studies for inclusion and consulted the reference lists of previously published primary and secondary papers to manually search for additional relevant papers. Any disagreement regarding eligibility and inclusion in the systematic review was resolved through discussion and consensus between the two readers. If consensus was not reached, the opinion of a third author (RM) who acted as a “blind” final arbiter was requested. Investigators and corresponding authors were contacted for studies with incomplete data in order to obtain additional information if needed.

## 3. Results

### 3.1. Literature Search

The literature search strategy yielded 673 references (301 in PubMed, 371 in Trip and one identified through manual search).

**Figure 1:**
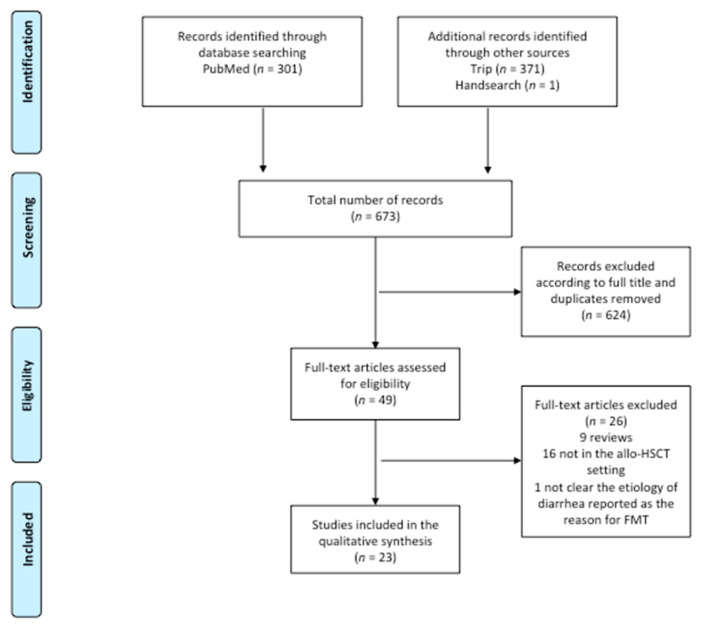
Preferred Reporting Items for Systematic Reviews and Meta-Analyses (PRISMA) flow diagram of the search strategy and included studies. The relevant number of papers at each point is given.

As shown in Figure 1, the number of potentially relevant papers identified by titles was 49. Among these 49 studies, 25 were excluded from the systematic review because they were reviews or did not address the role of FMT in the allo-HSCT setting. One paper was excluded because the etiology of diarrhea, reported as the reason for FMT, was not clear [32]. Of the 23 studies assessed, 15 were case reports or retrospective case series [30,33,34,35,36,37,38,39,40,41,42,43,44,45,46], seven were prospective cohorts [47,48,49,50,51,52,53], while only one completed randomized controlled trial was found in the literature [54].

In the following sections, we will present evidence on the use of FMT in allo-HSCT recipients. In the papers included in this qualitative synthesis, FMT was performed either with a therapeutic aim, both in the context of rCDI and as a second-line agent for gut aGvHD, or as a preventive strategy, in order to reduce dysbiosis or decolonize from ARB. A brief overview of the risks of FMT reported in the literature in this peculiar population will also be provided.

### 3.2. rCDI

Five studies evaluated FMT for the treatment of allo-HSCT recipients with rCDI. Neeman et al. and De Castro et al. first reported two successful case reports, in which FMT was performed by injecting fecal material via a nasojejunal tube from a related donor, or with material from two different donors delivered by means of push enteroscopy [39,40].

Since then, three small series have been published. Webb et al. analyzed seven allo-HSCT recipients who underwent FMT via nasojejunal tube or colonoscopy from an unrelated donor, with five of these patients still under immunosuppressive therapy. Six patients had no relapse, while one needed another FMT to obtain remission [41]. Another series reported FMT administration in three pediatric patients from related and unrelated donors via a gastric tube or colonoscopy, with only one achieving rCDI remission [42]. Moss et al. delivered FMT to eight patients as oral encapsulated therapy from unrelated donors. Resolution from rCDI was achieved in all patients at eight weeks, and only one had a recurrence at a later time. A metagenomic analysis of the stools showed a modification of the gut resistome (i.e., the set of genes conferring antibiotic resistance in the GM), with a reduction in the burden of antibiotic resistance genes by >50% following FMT, which persisted for more than one year. Conversely, the analysis of the dynamics of microbial communities highlighted the limited durability of the specific bacterial consortium introduced with FMT, with short-term similarity and long-term dissimilarity between donor and recipient GM composition [43].

### 3.3. Steroid-Resistant Gut aGvHD

Nine papers explored FMT as a potential therapeutic strategy for steroid-resistant or steroid-dependent gut GvHD (Table 1), defined as progression within 3–5 days or incomplete response by 7–14 days of treatment (steroid resistance) or recurrence after initial dose reduction (steroid dependence) [5].

Kakihana et al. reported for the first time the use of FMT in patients with steroid-resistant or dependent gut aGvHD [45]. They administered FMT from a related donor by nasoduodenal tube in four patients. All patients responded, with three complete responses and one partial response, but in three cases a second FMT was needed. Improvement of the gastrointestinal symptoms was observed within several days, combined with an increase in peripheral effector regulatory T cells [47].

In a subsequent report, three patients received FMT delivered by colonoscopy for refractory grade IV gut GvHD from related and unrelated donors. A clinical response with stool volume reduction was observed in all patients. Two achieved complete response with multiple FMT after 73 and 29 days from the first fecal infusion, while the other obtained a partial response, still presenting with grade I GvHD after one course of FMT. Based on 16S rRNA gene analysis of the pre- and post-FMT GM, restoration of microbial diversity and richness correlated with clinical improvement [44].

Another study involved eight patients with refractory grade IV gut GvHD receiving one or two courses of FMT from unrelated donors via a nasoduodenal tube. Symptoms were relieved in all patients, and five of them experienced complete response and no relapse. One week after FMT, the GM analysis of patients showed improved bacterial diversity and enrichment in health-promoting taxa, particularly *Bacteroides* and *Ruminococcaceae* [48].

Von Lier et al. reported 15 patients who received a single FMT via nasoduodenal infusion from an unrelated donor. A total of 10 patients showed complete remission within one month after FMT, without additional interventions to alleviate GvHD symptoms. In six of them, immunosuppressant drug therapy was successfully tapered within six months. In the other four individuals undergoing a complete response, GvHD symptoms returned upon the tapering of immunosuppressive therapy. The positive clinical response was accompanied by an increase in GM alpha diversity and partial engraftment of donor bacterial species. Moreover, increased relative abundance of short-chain fatty acid-producing bacteria, including Clostridiales members and particularly *Blautia*, was observed in the recipient’s stool [49].

Two other cases of allo-HSCT patients receiving multiple FMT from unrelated healthy donors via a nasogastric tube were reported. One patient experienced complete remission of gastrointestinal symptoms, but died more than one month later due to liver aGvHD and bloodstream infections related to the indwelling catheter. The other had only a temporary reduction in symptoms; diarrhea recurred one week after the last FMT and the patient died from multiorgan failure [45].

Kaito et al. reported the first case in which FMT from a related donor was performed by the administration of oral capsules in a patient with refractory gut GvHD [46]. A subsequent case series enrolled seven patients who received one to three FMT from unrelated donors, administered orally by capsules. After FMT, the introduction of new bacteria and an increase in microbial diversity was found in the recipient’s stool, with a strong reduction in the rate of bacterial dominance. Only two patients achieved complete remission, and one a partial response [50]. In the case report by Mao et al., after two cycles of oral FMT capsules from unrelated donor, intestinal aGvHD was gradually controlled and did not recur during the two-month follow-up. The diversity and structure of the GM after FMT were closer to those of healthy donors. Moreover, the amount of *Blautia* in the GM increased after FMT, which may explain the clinical improvement. Consistent with Kaito’s report, repeated doses of FMT brought continuous improvement of the gastrointestinal aGvHD symptoms. In this case, the symptoms improved but recurred after the first course of capsule FMT, while the second dose was effective in achieving complete remission [33].

The first report of FMT for refractory aGvHD in children was provided by Zhong et al. in 2019.

FMT was performed twice via a nasojejunal tube from an unrelated donor, and resulted in symptom remission. Taxonomic analysis of GM showed gradual reduction in Proteobacteria and increase in Firmicutes after FMT, and the restoration of diversity [34].

### 3.4. FMT as a Preventive Strategy

FMT was used as a preventive strategy in allo-HSCT in seven studies. In five of them, the aim was to decolonize from ARB strains, while in the other two the aim was to prevent and reduce GM dysbiosis (Table 2).

Bilinski at al. examined patients with blood disorders (40% neutropenic patients, 16% patients with aGvHD and 8% with chronic GvHD) colonized with ARB who underwent FMT via a nasoduodenal tube from unrelated donors; 60% of patients achieved complete decolonization one month after FMT [51]. Innes et al. described a case in which FMT was performed before HSCT to extensively eradicate drug-resistant organisms [35]. After these two encouraging reports, ten adult patients colonized by multidrug-resistant strains received FMT after (*n* = 6) or before (*n* = 4) HSCT from related or unrelated donors, delivered via enema or nasogastric tube. Three patients needed a second transplant from the same donor due to the initial failure of the procedure. Decolonization was achieved in seven out of ten patients. Interestingly, one case of grade III gut aGvHD still occurred after FMT performed before HSCT [36]. Ghani et al. delivered FMT from unrelated donors using a nasogastric tube in eleven patients with an underlying hematologic disorder, colonized by multidrug-resistant bacteria, of which eight underwent allo-HSCT after FMT. Although only 41% of patients were no longer colonized on rectal screening following FMT, there was a significant reduction in bloodstream infections by resistant and nonresistant strains compared to the control group. Moreover, shorter inpatient stays and fewer days of carbapenems administration were observed. Interestingly one patient developed bacteremia caused by a multidrug-resistant strain, but different from the previous colonizing microorganisms, and was treated effectively with a shorter antibiotic course [52].

Merli et al. carried out the only study with this aim in the pediatric population. They performed one course of FMT via esophagogastroduodenoscopy in five pediatric patients before allo-HSCT using samples from the same donor, to induce ARB decolonization. Eighty percent of patients tested negative for ARB strains within one week from FMT, but long-term decolonization was not achieved in four out of five patients [37].

Two other studies addressed FMT use to prevent and reduce dysbiosis. Autologous FMT after allo-HSCT was performed in a randomized controlled clinical trial. Compared with the control group, 16S rRNA gene sequencing of 14 patients after FMT revealed boosted microbial diversity and reestablishment of the GM composition they had before antibiotic treatment and allo-HSCT. In particular, important commensal groups, typically dominant in a healthy-like adult GM, such as *Lachnospiraceae*, *Ruminococcaceae* and Bacteroidetes members, were successfully re-established. According to a metagenomic analysis, auto-FMT also appeared to have reversed alterations in the functional content of the GM, mainly regarding genes involved in microbial virulence and metabolism [54]. In a subsequent analysis, they observed, during the first 100 days after engraftment, higher counts of neutrophils, lymphocytes and monocytes in the peripheral blood of auto-FMT recipients [12].

A similar result was obtained using FMT from unrelated donors, administered orally in 13 patients in the period immediately after neutrophil engraftment. FMT resulted in improved GM diversity associated with expansion of stool-donor taxa, including Clostridiales, and increased urinary levels of the tryptophan metabolite 3-indoxyl sulfate, recently proposed as a marker of GM eubiosis, associated with favorable outcome after allo-HSCT [55]. Notably, the subset of patients who received broad-spectrum antibiotics appeared to have attained the largest gains in terms of GM diversity. Two patients subsequently developed grade III–IV gut aGvHD, with one of them presenting concurrent bacteremia and subsequent multiorgan failure. There were no additional cases of bloodstream infections after FMT, but one case of CDI was observed [53].

### 3.5. Safety Issues in Allo-HSCT Recipients

The majority of the included studies report FMT as a generally well tolerated procedure, with no serious adverse events [32,33,34,35,36,37,39,40,41,42,43,44,45,46,47,48,49,50,51,52,54]. Interestingly, in the case series of Shouval et al. two patients developed bacteremia after the infusion, but targeted metagenomic sequencing demonstrated that the bacterial strains did not originate from the FMT inoculum [50]. DeFilipp et al. observed only one serious treatment-related adverse event (grade three abdominal pain) that resolved within 24 h of capsule administration [53]. Two studies specifically addressed the risks of FMT in allo-HSCT recipients. One patient enrolled in a trial to preemptively administer FMT oral capsules before allo-HSCT developed febrile neutropenia eight days after the last FMT dose, and died from severe sepsis two days later. The final results of blood cultures showed an extended-spectrum beta-lactamase-producing *E*. *coli* strain. The same strain was found in the lots of capsules from the donor, with a similar, but not identical, resistance pattern. Fecal samples of the recipient before FMT were negative for extended-spectrum beta-lactamase-producing microorganisms. Genomic relatedness between samples taken from the donor and blood cultures was calculated by means of whole-genome sequencing and single-nucleotide polymorphism-based analysis, and revealed that the bacterium was transmitted through FMT [30]. In another report, FMT was performed to decolonize from ARB before allo-HSCT. After ten days from allo-HSCT, Norovirus gastroenteritis was diagnosed, and it was later complicated by aGvHD. The symptoms resolved after a course of steroids and a second FMT from another donor with Norovirus-free stools. Fecal samples from the first FMT were analyzed and found to contain genotype II Norovirus, the same type identified in the patient’s stool. The authors speculated that Norovirus-induced colitis damaged the intestinal mucosa and “exposed” host antigens. Combined with increased gut permeability to molecules of a dysbiotic GM and an inflammatory milieu, this led to sensitization of allo-reactive lymphocytes and triggered aGvHD [38].

## 4. Discussion

In this systematic review we summarized the present literature on the use of FMT in the allo-HSCT setting. The role of FMT in the treatment of rCDI is established [24], and can be considered effective also in patients undergoing allo-HSCT [56]. Numerous studies evaluated FMT as a treatment for steroid-resistant gut aGvHD, providing encouraging preliminary data regarding feasibility and efficacy that need to be confirmed in larger prospective studies. However, diagnosis of aGvHD was not documented by biopsies in all cases in five studies [34,45,47,48,50], and this may have led to the inclusion of patients with other causes of diarrhea. The necessity of histological confirmation should be carefully taken into account in designing future studies, considering the invasiveness of the procedure especially in pediatric patients.

Moreover, steroid-resistant aGvHD carries a dismal prognosis, and the role of FMT in holding down the allo-immune response and improving survival could be less effective in patients with an already deteriorated clinical status and deeply altered GM and gut mucosa [28]. For this reason, FMT has been proposed to prevent the unavoidable dysbiosis occurring after HSCT and potentially reduce the incidence of GM-related complications, such as aGvHD and infections [57,58]. However, designing a study with the aim of improving gut eubiosis is challenging because it is difficult to evaluate efficacy while there are still no clear clinical and GM-related endpoints to assess [14].

The results of FMT in decolonizing patients from ARB are also promising, but the rates of decolonization vary from 20% to 70%.

Proposed mechanisms by which FMT can mediate clinical benefits include direct competition of the commensal microbiota delivered by FMT with pathogens, restoration of secondary bile acid and short-chain fatty acid metabolism, repair of the gut barrier and modulation of the mucosal and systemic immune system. However, further studies are needed to fill the gap in the comprehension of the exact mechanisms underlying FMT action [25,59].

**Figure 2:**
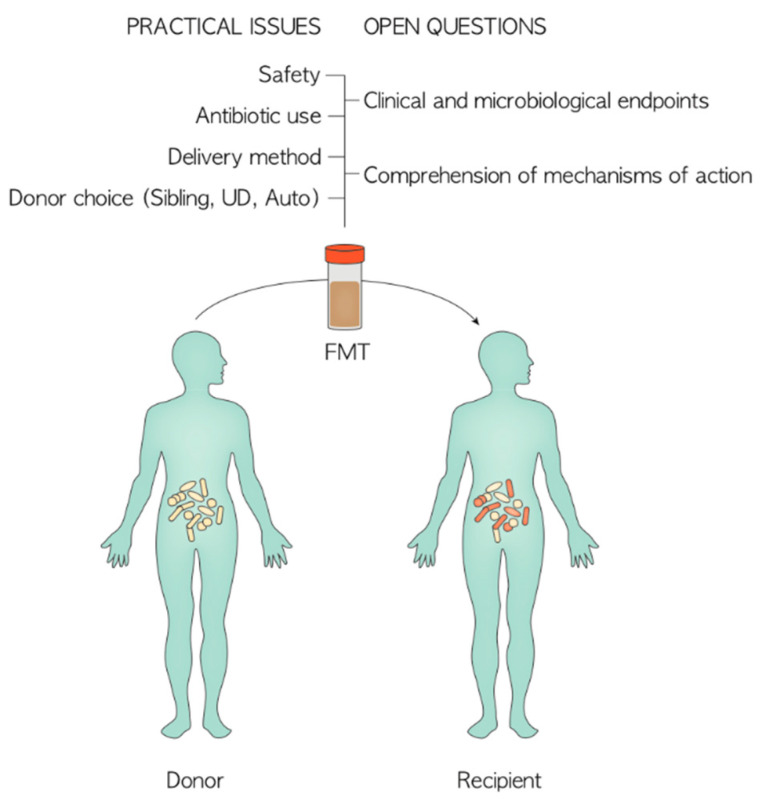
Practical issues and open questions to address in the design of future FMT intervention. UD: Unrelated Donor.

Another primary issue that should be addressed while discussing the results of FMT is the heterogeneity of key practical aspects that could influence clinical effectiveness, namely donor type, timing of infusion, delivery mode, stool screening, number of infusions and antibiotic policy (Figure 2). For example, to date no study exists in the literature comparing the results of related vs. unrelated donors of fecal material [60]. The use of frozen capsules from unrelated donors instead of siblings could reduce costs and waiting times, and consent a broader screening of fecal material, but they may be difficult to administer due to mucositis in the early phase after allo-HSCT [29,46]. Autologous FMT may also have the advantage of simple preparation and control during donor procedures, as well as reducing the risk of potentially transmitting pathogens from a third-party donor GM. However, a baseline healthy sample may not always be available. [11,54]. Repeated FMTs could increase the chance of durable modification of the gut ecosystem, thus improving the long-term achievement of clinical outcomes [37]. Antibiotics practice also influences the outcome of the procedure. Some studies discontinued them prior to the FMT procedure for a variable amount of time and routinely administered no specific pre-FMT antibiotic regimen, while others used oral colistin or vancomycin and neomycin before FMT to improve decolonization efficacy [35,37]. The use of antibiotics after FMT could also have a major impact. In the study by Val Lier et al., all the patients with secondary failure after a complete remission of gut aGvHD received antibiotics shortly after donor FMT, and the authors speculated that this may have interfered with a lasting response [49]. Bilinski et al. observed that patients who did not receive antibiotics within seven days after FMT achieved complete decolonization in a significantly higher proportion compared with those who did receive antibiotics [51].

Considering the risk of life-threatening infections in immunocompromised patients, antimicrobial treatment is pivotal, and the decision to withhold antibiotic therapy for any period of time to allow successful engraftment of the transplanted GM should be approached with caution. The relationship between timing of antibiotic course and FMT outcomes must therefore be a focus of research in the near future.

Despite the strong scientific rationale and the emerging potential clinical utility of FMT in allo-HSCT patients, the risk of infections resulting from the delivery of living microbial consortia to an immunocompromised host with impaired gut permeability must be of utmost concern [29]. While most studies deem FMT as a safe procedure in allo-HSCT recipients, some reports raised the concern of potentially transmitting pathogens from the fecal donor to recipients. This must prompt better efforts in extending donor stool screenings to rule out all potentially transmittable pathogens. Furthermore, correlating adverse events to FMT in these patients affected by multiple comorbidities is sometimes a difficult task, and advanced microbial analysis may be necessary [30,50]. The few data on the use of FMT in pediatric populations could raise specific concerns regarding the possible transmission of disease-related GM configurations and the long-term effects of GM manipulation [61,62], such as the occurrence of weight gain [63], irritable bowel syndrome [64] or long-lasting colonization by ARB [30].

## 5. Conclusions and Future Directions

FMT is a promising and potentially useful strategy with different purposes in allo-HSCT recipients. The microbiome could therefore be considered as a target of new individualized therapies, potentially guiding therapeutical decisions in the near future based on the patient’s GM signature. This fits into the medical model of personalized medicine, which stratifies people into different groups—with medical decisions, practices, and interventions being tailored to the individual patient based on the predicted response or risk of disease.

There is still much work to be done to understand if FMT can be implemented in clinical practice, both in terms of effectiveness and safety. Biological studies should provide novel insights into the comprehension of the mechanisms underlying the clinical findings. In particular, metagenomic and metabolomic analysis could help us to understand the effect of the administration of complex microbial consortia on the damaged gut ecosystem.

From a practical point of view, FMT should be performed in a selected center equipped with the required facilities to store, analyze and deliver the fecal material. A committed multidisciplinary team comprising hematologist, gastroenterologist, microbiologist, infectious disease physician and trained nurse is required to address the clinical complexity of the procedure.

Larger clinical trials are needed to definitively address the safety and effectiveness of this procedure for different purposes, and to define the main determinants of clinical response to FMT, such as the recipient’s basal GM layout and donor GM composition, antibiotic practice and the immune status of the host [65,66].

## Figures and Tables

**Table 1:** Summary of included studies regarding FMT as a therapeutic strategy for steroid-refractory or dependent gut aGvHD.

First Author	Year	Number of Patients	Route of Administration	Donor	CR	PR	CR/Patients %	CR + PR/Patients %	Comments
Kakihana	2016	4	Nasoduodenal tube	Relative or Spouse	3	1	75%	100%	Response assessed within 7–14 days; in three cases a second FMT was needed.
Spindelboeck	2017	3	Colonoscopy	Unrelated or Related	2	1	67%	100%	Two patients achieved complete response with multiple FMT, one obtained a partial response after a single FMT with persistent grade I GVHD
Qi	2018	8	Nasoduodenal tube	Unrelated	5	1	63%	75%	The FMT recipients exhibited improved progression-free survival within 90 days after the diagnosis, compared with an historical control group, but no difference in overall survival.
Kaito	2018	1	Oral capsules	Related	-	1	-	100%	Digestive symptoms improved soon after initiation of FMT. aGvHD improved to stage 1 after the second cycle of FMT with the improvement of endoscopic findings.
Shouval	2018	7	Oral capsules	Unrelated	2	1	29%	43%	-
Zhong	2019	1 child	Jejunal tube under gastroduodenoscopy guidance	Unrelated	1	-	100%	100%	-
Biernat	2020	2	Nasogastric tube	Unrelated	1	1	50%	100%	In one case complete remission was achieved, but the patient later died due to liver aGvHD and bloodstream infections. In the second case only temporary reduction and death occurred by multiorgan failure.
Mao	2020	1	Oral capsules	Unrelated	1	-	100%	100%	Complete remission after the first cycle of FMT. Recurrence 11 days later, but remission achieved with a second cycle.
Von Lier	2020	15	Nasoduodenal tube	Unrelated	10	-	67%	67%	Response assessed at 28 days after FMT. In six of the 10 complete responders, immunosuppression was successfully tapered within six months. In the other four, GvHD symptoms returned upon tapering of immunosuppressive therapy
**Total**	**-**	**42**	**-**	**-**	**25**	**6**	**60%**	**74%**	-

aGvHD: Graft versus Host Disease; allo-HSCT: allogeneic hematopoietic stem cells transplantation; CR: Complete Response; FMT: Fecal Microbiota Transplantation; PR: Partial Response.

**Table 2:** Summary of included studies regarding FMT as a preventive strategy in allo-HSCT patients.

First Author	Year	Indication	Number of Patients	Route of Administration	Donor	Main Results
Bilinski	2017	ARB decolonization	20 with blood disorders (10 neutropenic, 4 aGvHD, 2 chronic GvHD)	Nasoduodenal tube	Unrelated	60% of patients achieved complete ARB decolonization at one month after FMT.
Innes	2017	ARB decolonization	1 before allo-HSCT	Nasogastric tube	Unrelated	By day +16 after FMT, no ARB was detected on rectal screening swabs.
Taur	2018	Dysbiosis reduction	25 (14 received FMT; 11 control group with no intervention)	Enema	Autologous	FMT patients had boosted microbial diversity and reestablishment of the intestinal microbiota composition they had before antibiotic treatment and allo-HSCT.
DeFililpp	2018	Dysbiosis reduction	13	Oral capsules	Unrelated	Improved intestinal microbiome diversity associated with expansion of stool-donor taxa.
Battipaglia	2019	ARB decolonization	10 (6 before and 4 after allo-HSCT)	Enema or nasogastric tube	Unrelated or Relative	Decolonization was achieved in 7 out of 10 patients.
Merli	2020	ARB decolonization	5 children before allo-HSCT	Esophagogastroduodenoscopy	Unrelated	Long-term decolonization was not achieved in four out of five patients.
Ghani	2020	ARB decolonization	11 with blood disorders (8 before allo-HSCT)	Nasogastric tube	Unrelated	Decolonization in 41% of patients. Reduction in bloodstream infections.

ARB: antibiotic-resistant bacteria; allo-HSCT: allogeneic hematopoietic stem cells transplantation; FMT: Fecal Microbiota Transplantation.
